# Verifying a Solver for Linear Mixed Integer Arithmetic in Isabelle/HOL

**DOI:** 10.1007/978-3-030-55754-6_14

**Published:** 2020-07-08

**Authors:** Ralph Bottesch, Max W. Haslbeck, Alban Reynaud, René Thiemann

**Affiliations:** 8 NASA Ames Research Center, Moffett Field, CA USA; 9 SRI International, Menlo Park, CA USA; 10 KBR Inc., NASA Ames Research Center, Moffett Field, CA USA; 11 NASA Ames Research Center, Moffett Field, CA USA; 12 University of Innsbruck, Innsbruck, Austria; 13 ENS Lyon, Lyon, France

**Keywords:** Branch-and-bound, Isabelle/HOL, Linear programming, Polyhedra, Simplex algorithm

## Abstract

We implement a decision procedure for linear mixed integer arithmetic and formally verify its soundness in Isabelle/HOL. We further integrate this procedure into one application, namely into CeTA, a formally verified certifier to check untrusted termination proofs. This checking involves assertions of unsatisfiability of linear integer inequalities; previously, only a sufficient criterion for such checks was supported. To verify the soundness of the decision procedure, we first formalize the proof that every satisfiable set of linear integer inequalities also has a small solution, and give explicit upper bounds. To this end we mechanize several important theorems on linear programming, including statements on integrality and bounds. The procedure itself is then implemented as a branch-and-bound algorithm, and is available in several languages via Isabelle’s code generator. It internally relies upon an adapted version of an existing verified incremental simplex algorithm.

## Introduction

The computational problem of deciding whether a system of linear inequalities with integer coefficients has an integral solution arises in many practical situations. Since it is NP-complete, no currently known algorithm can in general avoid searches of exponential length. Furthermore, while satisfiable instances always have short solutions that can be efficiently checked, there need not be short, efficiently-checkable proofs for the fact that an instance is unsatisfiable, unless $\textsc {NP}=\textsc {co-NP}$. (Contrast this with the related problem of deciding whether a system of linear inequalities with integer coefficients has a *rational* solution – this problem is in P, and Farkas’ lemma provides a short and efficiently checkable certificate that an unsatisfiable instance indeed has no solution.) Thus, if a solver declares that a given instance is unsatisfiable over the integers, the length of any proof for this fact may be exponential in the size of the input instance, in which case the computational effort required to check such a proof would be exponential as well.

Instead of repeatedly performing certification tasks that require immense amounts of data and computational effort, it may be more fruitful to formally verify the soundness of a solver *once*, so that it can then be trusted without instance-by-instance certification of its output. The implementation of such a solver, together with a formal proof of its soundness, is the goal of the present work. Specifically, we use Isabelle/HOL [19] to implement and prove the correctness of a branch-and-bound algorithm [21, Chapter 24.1], and then use Isabelle’s code generator [11] to obtain verified executable code. Along the way, we also give the first formalized proofs for several important results on integer programming.

A concrete example of an application for our solver comes from *termination analysis*, where a program is given as input to a termination tool that tries to determine whether the given program terminates on all inputs. Since termination tools get patched and improved repeatedly, maintaining an up-to-date formal proof of soundness would be extremely difficult. Therefore, the approach that is typically used is to have the (unverified) termination tool output a certificate for its analysis, which can then be checked by a verified certificate checker. One such certificate checker is CeTA [5, 24]. It has been verified in Isabelle/HOL, so that whenever it accepts a proof of termination for some program, the formal proof of CeTA ’s soundness ensures that the program does indeed terminate.

As an example, consider a program to compute the binary logarithm.
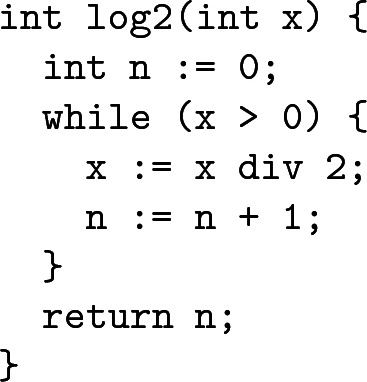


This program can be translated into an integer transition system and termination can be proved by showing that the value of x is decreased by at least 1 in every loop iteration. This property can be expressed in linear integer arithmetic (LIA): it is equivalent to the validity of formula (1), where $x'$ and $n'$ represent the new values of *x* and *n*, respectively, after an iteration of the loop.

1 $$\begin{aligned} x > 0 \wedge 2x' \leqslant x \wedge x \leqslant 2x'+1 \wedge n' = n + 1 \longrightarrow x \geqslant x' + 1 \end{aligned}$$

Validity of (1) is equivalent to unsatisfiability of the negated formula, which is simply a conjunction of linear inequalities:

2 $$\begin{aligned} x > 0 \wedge 2x' \leqslant x \wedge x \leqslant 2x'+1 \wedge n' = n + 1 \wedge x < x' + 1 \end{aligned}$$

A *sufficient* condition for the unsatisfiability of (2) over the integers (LIA) is the unsatisfiability of the same system over the rationals (LRA); the latter can be shown, for instance, via the simplex algorithm [9]. Indeed, a verified implementation [23] of the simplex algorithm is currently integrated into CeTA  [5]. However, whereas (2) is unsatisfiable over the integers, it has a rational solution $x = n' = 1$, $x' = \frac{1}{2}$, $n = 0$. For such examples, considering the problem over the rationals may prohibit CeTA from detecting unsatisfiability over the integers.

Therefore, in this paper we develop a verified theory solver for LIA (in fact, for linear *mixed* integer arithmetic, where only a user-specified part of the solution is required to be integral). The verified solver takes a conjunction of strict and non-strict linear inequalities as input, and decides whether they are simultaneously solvable. We fully integrate the LIA solver into CeTA, so that the new version can handle all instances that are unsatisfiable over the integers and not only those that are unsatisfiable over the rationals as well. Of course, the LIA solver can also be used as a stand-alone theory solver, e.g., to perform verified SMT solving.

We verify our LIA solver in two major steps.

1. First, we show that for every set of LIA constraints it suffices to search for small solutions. To this end, we formally verify an a priori bound in the style of Papadimitriou [20]: If there is an integer solution to a set of LIA constraints, then there is also one that is bounded by $b := n(ma)^{2m+1}$, where *n* is the number of variables, *m* the number of inequalities, and *a* the largest absolute value of any number occurring in the inequalities. To be more precise, the small solution satisfies $|x| \le b$ for each variable *x*.
  Our verified upper bound matches the one given in a textbook [21, Thm. 17.1] (which is considerably lower than the one by Papadimitriou).^1^ Specifically, we establish a bound of $(n+1)!a^n$ (with no dependence on *m*). To prove this bound in Isabelle/HOL we mostly follow the textbook proofs and formalize several important results from linear programming, often with additional statements on bounds and integrality. These results include: the fundamental theorem of linear inequalities, the Farkas–Minkowski–Weyl theorem, Carathéodory’s theorem, and the decomposition theorem for polyhedra. Note that the bound on the size of solutions also implies the fact that the problem of deciding satisfiability for linear integer inequalities is in NP.
2. Using the upper bound, we can decide satisfiability via a finite search. For instance, for formula (2) we have $n = 4$, $a = 2$ and $m = 6$ (the equality counts as two inequalities), and we know that if (2) is satisfiable, then there is an integer solution with absolute values at most 1920.
  To perform this search, we implement and verify a basic branch-and-bound algorithm. It is based on an incremental version of the simplex algorithm by Dutertre and de Moura  [10], which is used to deliver candidate solutions and to prune the search tree by detecting unsatisfiability in LRA. Although the incremental simplex algorithm has recently been verified in Isabelle/HOL [3], its integration into the branch-and-bound algorithm is not immediate: the branch-and-bound algorithm requires frequent updates of bounds on variables, and this operation is not supported by the existing verified incremental simplex algorithm.

Note that our verified LIA solver is missing several possible optimizations [6, 7, 14], some of which might be integrated in future work. Therefore, it clearly cannot compete with state-of-the-art (unverified) solvers. Still, our experimental results show that there are some examples from SMT-LIB where our solver is successful, but both CVC4 [2] and Z3 [16] fail.

**Structure.** We give some preliminaries on linear (integer) programming and Isabelle in Sect. 2. Afterwards, we present our formalization of linear programming and the mentioned bound in Sect. 3. The branch-and-bound algorithm with the adaptation of the incremental simplex algorithm are covered in Sect. 4. We provide experimental results in Sect. 5 and conclude in Sect. 6.

The collection of theorems on polyhedra and small solutions is available as part of the archive of formal proofs (AFP) in the entry on linear inequalities [4], and the branch-and-bound algorithm is part of IsaFoR/CeTA [24]. All of the theorems of this paper are linked to the formalization on an accompanying website. It also provides details on the experiments.

http://cl-informatik.uibk.ac.at/software/ceta/experiments/lia/

**Related Work.** Allamigeon and Katz [1] have implemented the simplex algorithm in Coq and used it to give constructive proofs of a number of important theorems about convex polyhedra. The overlap between our work and [1] consists of formalizations of basic facts concerning cones and polyhedra, the fundamental theorem of linear inequalities, and Farkas’ lemma. However, whereas in [1] a simplex algorithm for optimization problems is implemented in order to be used in constructive mathematical proofs, we formalize theorems concerning integer programming, including bounds on the size of solutions, and use these together with the previously Isabelle-verified simplex algorithm to obtain formally verified, yet efficient, software.

There is also a formalization of theorems about polyhedra in HOL Light, due to Harrison [12], but it contains neither a formalization of the simplex algorithm nor does it cover integer programming.

Cooper’s algorithm has been formalized by Nipkow [18] in Isabelle/HOL. Although this algorithm also solves linear integer arithmetic, it internally works completely differently and its formalization requires different proofs; therefore, we do not see any overlap between the two works. We nevertheless consider the verified version of Cooper’s algorithm in our experiments.

Finally, we mention two general-purpose verified solvers. Carlier *et al.* [8] used Coq to implement and verify an algorithm for solving constraint satisfaction problems over finite domains. As with [1], the resulting implementation can be used in principle, but is not efficient enough to compete with unverified implementations of the same algorithm. Narkawicz and Muñoz [17] used PVS to verify a general branch-and-bound algorithm; a C++ implementation of this algorithm is described in [22]. In contrast to our work, this implementation was not automatically generated from a formal, verified algorithm specification, but was coded separately. Furthermore, in order to use the general branch-and-bound algorithm, one must first tailor it to an application domain by specifying a number of functions that must respect certain specifications, whereas every part of our LIA solver (both branch-and-bound and simplex) has been formally verified. Thus, while the algorithm we verify lacks the generality of the one in [17], our implementation retains a higher degree of reliability than the one in [22], due to being entirely generated from a formally verified algorithm, and it is nevertheless reasonably efficient.

## Preliminaries

We briefly review some linear programming and Isabelle background.

### Linear Programming

We assume familiarity with vector spaces. Although our Isabelle theorems use a more general type, here we present our results in the context of Euclidean spaces ($\mathbb {R}^n$). We denote the usual inner product in $\mathbb {R}^n$ by ‘$\cdot$’.

A *(non-strict) linear inequality* is an inequality of the form $a\cdot x \le b$, where $a,x\in \mathbb {R}^n$ (*a* a row vector, *x* a column vector) and $b\in \mathbb {R}$. A system of linear inequalities can therefore be written as $Ax\le b$, with $A\in \mathbb {R}^{m\times n}$ and $b\in \mathbb {R}^m$ a column vector. A system of linear inequalities is a *mixed integer system* if, for some $I\subseteq \{1,\ldots ,n\}$, it is required that $x_i\in \mathbb {Z}$ for all $i\in I$. We also define *strict* linear inequalities to be inequalities of the form $ax<b$, with *a*, *x* and *b* as before.

In this work we consider mixed integer systems of linear inequalities containing both non-strict and strict inequalities.

For reference, we collect below the definitions of several important concepts from linear algebra that are needed in order to state the theorems that we formalize. These definitions can be found in textbooks on linear programming such as [21, Chapters 7.1–2 and 16.2].

#### Definition 1

**(Half-spaces, hyperplanes, polyhedra).** For $c\in \mathbb {R}^n\setminus \{0_n\}$ (a row vector) and $d\in \mathbb {R}$, we say that the set $H = \{x \mid c\cdot x \le d\}$ is an *affine half-space*, and that *c* is its *normal vector*. If $d = 0$, then *H* is called a *linear half-space* (or just a *half-space*). The set $\{x \mid c \cdot x = 0\}$ is called a *hyperplane* (of which *c* is a normal vector).

A set $P\subseteq \mathbb {R}^n$ is called a *(convex) polyhedron* if $P = \{x\mid Ax \le b\}$, for some matrix $A\in \mathbb {R}^{m\times n}$ and $b\in \mathbb {R}^m$. In words, a polyhedron is the intersection of a finite collection of affine half-spaces.

#### Definition 2

**(Cones).** A non-empty set $C\subseteq \mathbb {R}^n$ is a *cone* if, for all $x,y\in C$ and $\lambda ,\mu \ge 0$, we have $\lambda x + \mu y\in C$. A cone *C* is *generated* by the set of vectors *X* if $C = \left\{ \lambda _1v_1+\ldots +\lambda _m v_m\mid \lambda _1,\ldots ,\lambda _m\ge 0,\{v_1,\ldots ,v_m\}\subseteq X\right\}$, and *C* is *finitely generated* if it is generated by a finite set of vectors. A cone is *polyhedral* if it is the intersection of finitely many (linear) half-spaces.

#### Definition 3

**(Convex hull, polytopes, integer hull).** The *convex hull* of a vector set *X* is the set of all convex linear combinations of vectors from *X*. More precisely,

$$\begin{aligned} \text {conv.hull }X = \{\lambda _1 v_1 + ... + \lambda _m v_m \mid \lambda _1,...,\lambda _m\ge 0, \sum \lambda _i = 1,\{v_1,...,v_m\}\subseteq X\} \end{aligned}$$

The convex hull of a finite set of vectors is called a *(convex) polytope*.

Finally, if *P* is a polyhedron, then the *integer hull* of *P*, denoted $P_{I}$, is the convex hull of the set of integral vectors of *P*. (Integral vectors are vectors whose coordinates with respect to the standard basis are integers.)

### Isabelle

For our formalization work we use the theorem prover Isabelle. Knowledge of Isabelle will be helpful, but is not necessary in order to read the paper, as we have tried to make the formal source listings accessible even to a reader with a purely mathematical background.

Nevertheless, we briefly explain the meaning of some important notation here. First, we have  ,  , and denote the zero-vector of dimension *n* by  . Often, the statement that a vector or a matrix has a certain property will be expressed as membership in the set of all vectors or matrices with that property:  is the set of vectors (of finite dimension) with entries bounded in absolute value by  (similarly  ),  is the set of vectors *v* with $v_i\in \mathbb {Z}$ for all  , and, finally, $\mathbb {Z}_{v}$ is the set of vectors (of finite dimension) with integer entries (similarly, $\mathbb {Z}_m$ is a set of matrices). We also have a notation for sets defined by some set of vectors or by a matrix:  denotes the cone generated by the finite set *X*; other examples are  ,  and  , all with the obvious meanings.

## Mixed-Integer Linear Problems

### The Main Formalized Theorems

We discuss our formalization of several results that are needed in order to formally prove the soundness of a branch-and-bound-based solver for mixed-integer linear systems of inequalities. The main theorem for this purpose states that if a mixed integer system of linear inequalities can be described using only integers, then it has a solution if and only if it also has a solution involving only numbers of bounded size.

#### Theorem 4


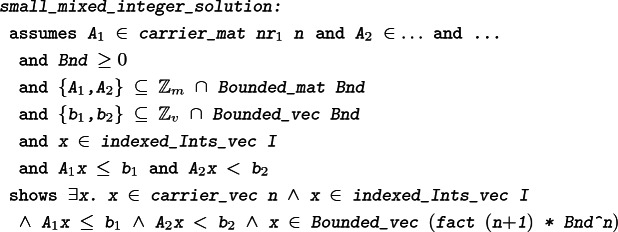


In order to derive this result, we require formalizations of several results from the theory of linear inequalities, beginning with the fundamental theorem of linear inequalities. This theorem states that for any finite set of vectors *A* and vector *b*, either *b* is in the cone generated by a subset of *A*, or there exists a hyperplane $\{x\mid c\cdot x=0\}$ separating *b* from *A* and containing some number of vectors of *A*.

#### Theorem 5


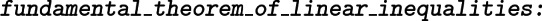
 2 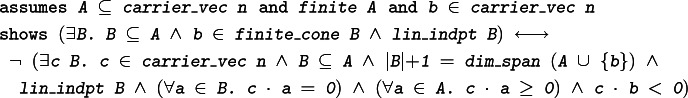


To prove the theorem, one first considers an algorithm that iteratively applies a procedure that takes a subset of vectors from *A* and produces either the cone containing *b* from the theorem statement, or the separating hyperplane, or a new set of vectors from *A*. In case of the third outcome, the output set is used as the input for the next iteration. Thus, starting from some valid set of vectors, the above algorithm either never terminates (if the third outcome occurs in every iteration), or it produces an object satisfying the theorem statement. The proof is completed by showing that an infinite execution cannot occur.

The above argument could in principle be formalized in Isabelle by defining a function that incorporates the algorithm, and then proving that the function is well-defined (which implies the termination of the algorithm on all inputs). However, we are really only interested in the algorithm’s termination; the fact that some input is mapped to a certain output is irrelevant for the proof of the theorem. Furthermore, we only need that the algorithm terminates when the set of input vectors is valid (i.e., of the right cardinality and linearly independent), but, due to the limitations of the Isabelle function-package [13], the domain of a function cannot be restricted in this manner. Consequently, we formalize the proof without modeling the algorithm as an Isabelle function. Instead, we define a relation on pairs of valid subsets of *A*: The pair $(J',J)$ is in the relation if and only if, starting with *J* as input, one iteration of the algorithm produces output $J'$. In other words, the relation encodes all iterations of the algorithm where the third outcome occurs. Since *A* is finite, termination is equivalent to the fact that the above relation has no cycles. The latter fact is established by a proof by contradiction (here, our formalization closely follows the textbook proof [21, Chapter 7.1]).

We also need to formalize three corollaries of Theorem 5. First, we have the theorem of Carathéodory, which follows directly.

#### Theorem 6


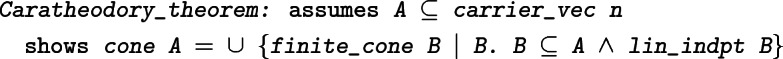


Next, we have the Farkas-Minkowski-Weyl theorem, which states that a cone is polyhedral if and only if it is finitely generated.

#### Theorem 7


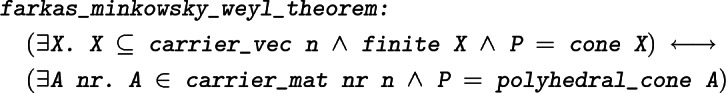


The proofs of Theorems 7 and 5 in [21] contain some simplifying assumptions that can be made without loss of generality. Of course, in Isabelle we must provide the full details of every proof, which often entails a non-trivial amount of additional formalization work. For example, the textbook proof of the “$\longrightarrow$”-implication of Theorem 7 only covers the case where *X* spans $\mathbb {R}^n$. One way to recover this part of the theorem in full generality is to identify the span of *X* with $\mathbb {R}^m$ for some $m < n$, apply the “$\longrightarrow$”-implication for dimension *m*, and then extend the half-spaces (of $\text {span }X$) that define the polyhedral cone, into $\mathbb {R}^n$. In fact, this argument is essentially the justification for the *wlog* that is given in the book. Unfortunately, the Isabelle vector/matrix library we use does not support identifying an arbitrary proper subspace of $\mathbb {R}^n$ with a Euclidean subspace of lower dimension: Even if we prove some statement for  , we cannot apply it to some arbitrary *m*-dimensional subspace of $\mathbb {R}^n$. Instead, our formalization of the general case involves adding suitable dummy vectors to *X* until the set spans all of $\mathbb {R}^n$, so that we can apply the full-dimension implication for  . This is one of several situations where filling in the “obvious” steps of a proof in a way that can be formally expressed in Isabelle requires some creativity.

The third corollary is the decomposition theorem for polyhedra, stating that every polyhedron can be written as the sum of a polytope and a polyhedral cone:

#### Theorem 8


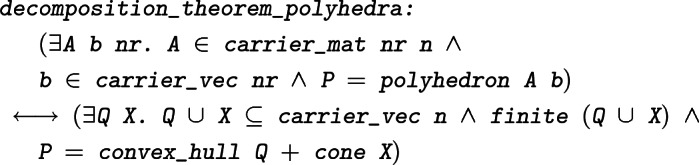


For both Farkas-Minkowski-Weyl (Theorem 7) and the decomposition theorem, the fact that we used a set-based matrix/vector library proved to be beneficial. To show the “$\longrightarrow$”-implication of Theorem 7, one defines a matrix, the dimension of which is a function of *X* (and can therefore not be independently fixed just by the type of *X*). Constructing matrices of dimensions that depend on the value of some variable is easy when using  , but would be very difficult with matrix libraries which utilize Harrison’s encoding of dimensions in types [12]. In the case of the decomposition theorem for polyhedra, the proof involves adding a new component to each vector from a set of *n*-dimensional vectors and then reasoning about the resulting set of $(n+1)$-dimensional vectors, while maintaining the correspondence between the two sets. Here, the use of  makes it possible to easily switch between dimensions and reason about objects such as “the vector formed of the first *n* components of some $(n+1)$-dimensional vector”.

Since the set of (real) solutions of a system of linear inequalities is a polyhedron, the decomposition theorem for polyhedra allows us to write any solution vector *x* as $y+z$, with *y* an element of a polytope (and therefore bounded), and *z* an element of a finitely generated cone. This suggests the following approach to proving Theorem 4 (  ): If *x* is such that $x_i\in \mathbb {Z}$ for all $i\in I$, we may try to replace *z* with a vector $z'$ of the same cone, with bounded entries, such that $(y+z')_i\in \mathbb {Z}$ for all $i\in I$ (thus, $y+z'$ would be the desired bounded solution). This approach does in fact work, but it clearly requires a more powerful version of the decomposition theorem, since the one we have shown so far says nothing about bounds or integrality. The proof of the new decomposition theorem also requires a bounded integer version of Theorem 7. This latter theorem in turn is based on a modified version of Theorem 5 which describes more precisely how separating hyperplanes can be computed so that the normal vectors are integral and with components of bounded size.

#### Theorem 9


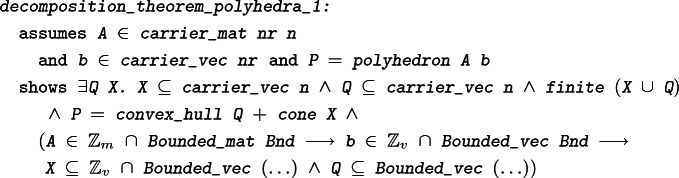


The ‘$\longrightarrow$’-implication of this stronger version of the decomposition theorem for polyhedra states that if *A* and *b* have bounded integer entries, then the finite sets *Q* and *X* can be chosen such that they contain only bounded vectors and, furthermore, such that *X* contains only integral vectors. The integrality of the vectors in *X* is the crucial ingredient necessary for constructing the vector $z'$ as required and completing the proof of Theorem 4.

In [21], only a weaker version of Theorem 4 is proved; it covers only the case of non-strict linear inequalities with integral solutions. Although our result trivially implies this weaker form, we have formalized the proof from the textbook as well, for the sake of completeness.

This proof relies on a decomposition theorem for the integer hull of a polyhedron, which also requires bounded integer versions of Theorem 7 and the decomposition theorem for polyhedra. Only a rough sketch is given in the book as to how the bounded integer versions of these theorems can be obtained. When formalizing this part, however, we encounter the following issue: In the course of a proof, it will be necessary to add new vectors to a set until it has a certain property, or to add half-spaces to a collection until its intersection coincides with some polyhedron. This suffices if we only wish to prove the *existence* of a set of vectors with some property, or of a specific representation of a polyhedron, but if we also need to prove bounds on the numbers needed to describe these objects, it becomes crucial *which* vectors or half-spaces are chosen, because some choices, while valid, will lead to results that do not respect the desired bounds.

For a concrete example, we return to the “$\longrightarrow$”-implication of Theorem 7 (Farkas-Minkowski-Weyl), this time in its bounded integer version:

#### Theorem 10


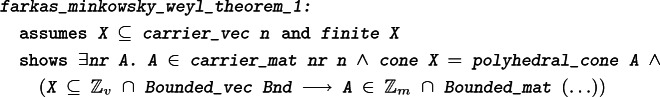


As mentioned earlier in this section, this implication is proved for the case where the span of *X* is $\mathbb {R}^n$, which is then used to prove the general implication, but the switch from the special to the general case involves adding vectors to *X* until the set spans the entire space, and then applying the full-dimension statement to obtain the half-spaces that define the polyhedral cone. Now, the vectors that are added to *X* can affect the size of the entries of the resulting matrix *A*, and the fact that these vectors can also be chosen in such a way that the entries of *A* are bounded in terms of only  and *n*, is not obvious, and in fact requires a careful construction. Whereas such matters are simply glossed over in the textbook, resolving the *wlogs* in the proof of the bounded version of Theorem 5 and of Theorem 7 resulted in Isabelle proofs of 176 lines and 110 lines, respectively.

In the end, we achieve the following formalized version of the textbook theorem [21, Thm. 17.1].

#### Theorem 11


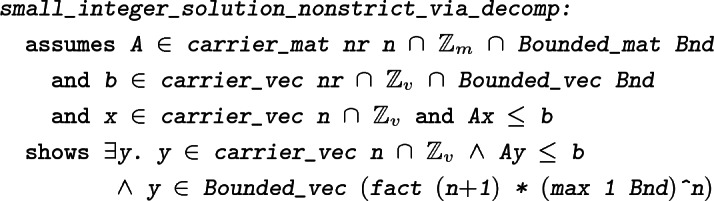


### Additional Formalized Theorems

In order to formalize the proofs of the main theorems, we collect a number of basic lemmas concerning cones, convex hulls, integer hulls, normal vectors and bases of vector spaces. On the one hand, these lemmas include very basic statements that would not normally require separate proofs, but were needed for the formalization, such as the fact that a set of vectors is a subset of the cone it generates, or that a convex combination of two vectors of a cone belongs to the cone. On the other hand, our supporting lemmas include statements that appear in standard mathematical texts, such as the fact stated in Lemma 12 that any linearly independent set of vectors can be extended to a basis of the vector space. We mention that we have proved all of these facts only for Euclidean vector spaces, making heavy use of the fact that the dimension is finite, because this case suffices for our application.

#### Lemma 12


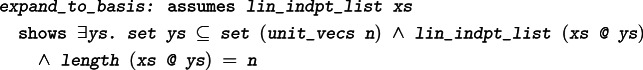


We note that in Lemma 12,  is list concatenation and  refers to the standard basis of $\mathbb {R}^n$. Of course, a linearly independent set can be extended in many other ways, but we use vectors from the standard basis because they allow us to obtain the same number bounds as for the original linearly independent set. Adding the standard basis vectors is also the reason for using  instead of  in many theorems that mention upper bounds. Indeed, the “  ”-operation often cannot be dropped. For instance, consider the “$\longleftarrow$”-implication of Theorem 7 and the degenerate case where the matrix *A* is empty or just contains zeros. Then the entries of *A* are bounded by 0 and the cone is the whole space. Thus, for generating this cone one needs at least *n* non-zero vectors, e.g., the unit vectors. And these do not have all their entries bounded by 0, but by  .

A notable exception, without “  ”, is our main Theorem 4 (  ). This result is first proved with the “  ” expression in the bounds. The version without the  -operation is then established by proving that the theorem also holds in all degenerate cases (where the bound is 0).

Aside from the main theorems and supporting lemmas, we also formally prove two variants of Farkas’ lemma. We do not need these for our work on the verified linear arithmetic solver, but obtaining them did not entail a prohibitively large additional effort, and they may be useful for other formalizations.

Although there already exists an entry for Farkas’ lemma in the AFP, its proof there is based not on the fundamental theorem of linear inequalities (Theorem 5), but on a separate formalization of the simplex algorithm (one that has been formalized solely for rational numbers). Since here we use Theorem 5, we obtain a version of a lemma that allows for the use of a more general type than just the rationals. (In Isabelle, type annotation is denoted by  . Below,  is a type variable that stands for the type of the entries of a matrix/vector; it can be any type with the suitable algebraic properties.)

#### Lemma 13


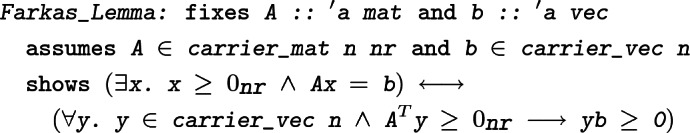


#### Lemma 14


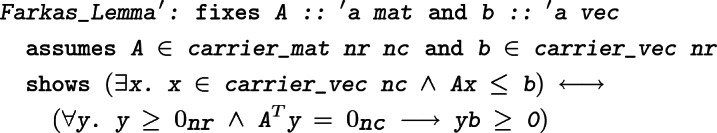


Finally, we remark that, while the first of the two variants of Farkas’ lemma follows easily from Theorem 5, the second variant (which, in [21], has a three-line proof that is based on the first variant) is somewhat more difficult to formalize. This is because its proof involves concatenating matrices and deducing inequalities involving the resulting matrix from facts about its components. Such operations require laborious low-level manipulations of vector inequalities, turning a three-line textbook proof into 102 lines of Isabelle code.

## A Verified Branch-and-Bound Algorithm

### The Branch-and-Bound Algorithm

Algorithm 1 shows the Isabelle/HOL function  , which is our implementation of a branch-and-bound algorithm for solving LIA problems. It takes as parameters a list of constraints  , the list of variables  that should get an integer assignment and (total) functions  and  that map the variables in  to their lower and upper integer bounds.  returns either a satisfying assignment which maps variables to rational numbers and all variables in  to integers, or  , if the mixed integer problem is unsatisfiable within the bounds  and  .  first uses the simplex algorithm to find a rational solution of the constraints within the bounds. If the constraints are already unsatisfiable in the rational numbers or if the solution is already integral for all values in  , then  terminates accordingly. Otherwise, there exists an  where  (the value assigned to  in the rational solution  ) is not an integer. We update the bounds on  once in  and once in  and branch by running  with the new upper bound and then with the new lower bound.

To verify  in Isabelle/HOL we have to show that it always terminates. Note that in every recursive call, we either decrease one of the upper bounds  or increase one of the lower bounds  . This fact is used to show that in every recursive call, the range of possible values decreases for *some*  , and, hence, so does the search space. Thus, we use the following measure (of the size of the search space) to prove termination in Isabelle/HOL: 
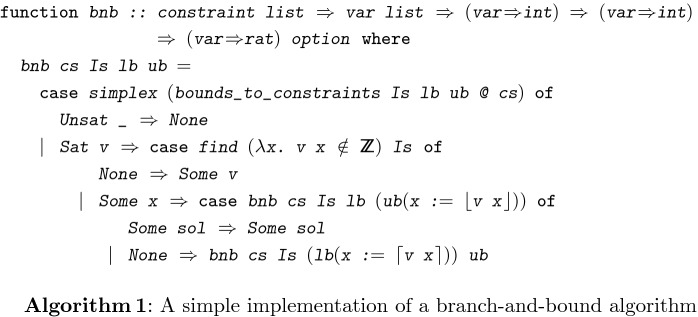

$$\begin{aligned} \max \left( 0, \sum _{x_i \in \mathtt {Is}} (\mathtt {ub}(x_i) - \mathtt {lb}(x_i))\right) \end{aligned}$$

We then prove two theorems about  : any detected solution is valid, and whenever  delivers  , no solution exists within the range that is specified by the lower- and upper-bounds. The expression  means that the solution  satisfies all of the constraints in  and that all  are assigned integer values by  . 
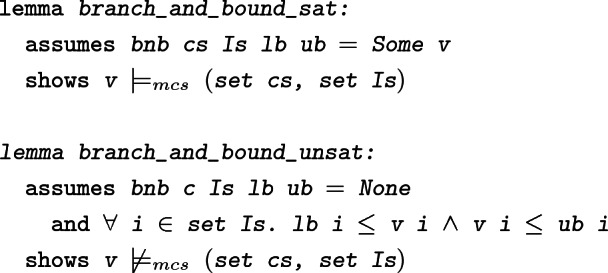
 At this point we connect the branch-and-bound algorithm with the bounds from Sect. 3 to obtain a decision procedure for linear (mixed) integer arithmetic: 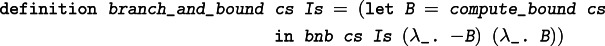


Here,  is an algorithm that extracts the relevant parameters (number of variables, maximal absolute value in constraints) and then calculates the upper bound as in Sect. 3. One complication comes from the fact that there are two different representations of constraints: the statements regarding bounds have been proved for constraints given in matrix-vector form, $Ax \le b$ or $Ax < b$ with integral matrix *A* and integral vector *b*, whereas the input to the branch-and-bound algorithm is a set of constraints, where each constraint is represented via a (sparse) linear polynomial with rational entries, e.g., $x_5 + \frac{1}{10}x_{1041} \le \frac{7}{3}$. Hence,  internally also normalizes the constraints, e.g., by canceling fractions, and by renaming the variables so that the indices of variables with non-zero coefficients form a contiguous block: $x_0,\ldots ,x_{n-1}$. The normalized constraints can then easily be translated into matrix-vector-form, which enables a lifting of Theorem 4 (  ) to constraints that are represented via sparse polynomials. 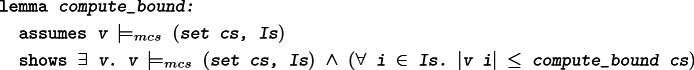


At this point, it is easy to combine the results of  with  to finally show that  is a complete and sound decision procedure. Either it returns some assignment, which is then a solution to the mixed integer problem; or it returns  , and the mixed integer problem is unsatisfiable. 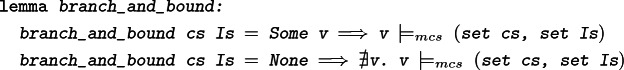


### Using the Incremental Version of Simplex

One problem of the branch-and-bound algorithm from the previous section is in the way it invokes the simplex algorithm: although in every iteration only a single constraint changes, the simplex algorithm is always started from scratch.

Therefore, in this section we optimize the branch-and-bound algorithm to use an already existing verified *incremental* version of the simplex algorithm [3, 15], which returns a state instead of only returning a satisfying assignment or stating unsatisfiability. The state contains for instance a tableau, i.e., a list of equations which is essential for the simplex algorithm. By reusing the state, expensive operations like creating the tableau can be avoided, making the incremental simplex very attractive to be used within the branch-and-bound algorithm.

A complication arises, since the verified incremental simplex algorithm was developed to be used in a DPLL(T)-solver, where all constraints are known beforehand and the constraints are not changed throughout one DPLL(T) run. Therefore, the incremental interface does not allow for changing constraints or adding new ones. As a consequence, an integration of the incremental simplex into the branch-and-bound algorithm is not immediate, since there the bounds are changed in every iteration.

Our solution is a slight extension of the incremental simplex algorithm. To be more precise, we write a function which changes exactly one constraint in the state in a way that the relevant invariants of the incremental interface still hold. This extension allows us to reuse all the existing soundness properties and proofs of the incremental simplex algorithm without modifications. It is specifically tailored for running the branch-and-bound algorithm. We choose this approach instead of adding a feature to change arbitrary constraints in the incremental simplex interface, since such a feature would require a major rewrite.

Since the algorithmic structure and the soundness statement of the modified branch-and-bound algorithm is completely identical to the one of Sect. 4.1, we just refer to the formalization for further details.

## Benchmarking

We tested two versions of our solver (based on incremental/non-incremental simplex) by comparing them with two well-established SMT-solvers, Z3 and CVC4. Testing was done on a subset of the non-incremental^3^ QF_LIA (quantifier-free linear integer arithmetic)^4^ benchmark set from SMT-LIB. For this experiment we had two goals in mind: 1. to see whether it is worthwhile to use the non-incremental version of simplex as a sub-routine in the branch-and-bound algorithm, and 2. to get an idea about the extent to which our verified, non-optimized solver can handle practical examples.

We did not go through the effort of making our solver compliant with the language of SMT-LIB, as we felt that for the above two goals, it would suffice to write a simple converter that could handle a reasonable portion of the QF_LIA benchmarks. Thus, we obtained a dataset of 1192 benchmarks, comprising 18% of the 6489 benchmarks in QF_LIA. (More specifically, the following sub-folders were fully converted to a format that is readable by our solver: 20180326-Bromberger, miplib2003, pb2010, pidgeons, prime-cone, and slacks.) All solvers were tested on this dataset, on the same hardware, with a 60s-timeout per benchmark. Z3 version 4.4.0pre-2, CVC4 version 1.5-4, and the 2019-05-09 release of SMT-LIB were used.

The only other verified LIA solver that we are aware of is an Isabelle formalization of Cooper’s algorithm in the AFP. This algorithm solves a more general problem than linear integer arithmetic (namely linear arithmetic with arbitrary quantifiers over integer variables). We obtained an implementation with minimal changes to make code generation possible (just as we produced executables for our own solver).

**Table 1.** Experimental results

	Sat	Unsat	Total
Non-incremental bnb	245	131	376
Incremental bnb	314	131	445
CVC4	470	158	628
Z3	570	164	734
Verified Cooper	2	0	2

*Evaluation.* Our branch-and-bound implementation solves 37% of the dataset with incremental simplex as a sub-routine, and 31% with non-incremental simplex (Table 1). Since we have only implemented a naive branch-and-bound algorithm, without any additional heuristics for pruning the search space, it is unsurprising that its performance cannot match that of more mature solvers. Somewhat surprising is the fact that some benchmarks are solved by our solvers but not by Z3 or CVC4: of the benchmarks solved by incremental bnb, 28 are not solved by Z3, 29 are not solved by CVC4, and 8 are solved by neither Z3 nor CVC4.

Interestingly, the non-incremental simplex-based solver can handle a few instances that the incremental simplex-based solver does not. Although using an incremental simplex leads to better overall results, it appears that reusing valuations from previous simplex runs can sometimes lead the search astray in such a way that simple solutions are missed. The phenomenon of a search proceeding in the wrong direction and missing a simple solution may also be the reason why some instances cannot be handled by either Z3 or CVC4, despite being solved by our solver.

Cooper’s algorithm is known to have a very high asymptotic complexity, which means that its performance is not a matter of optimizing an implementation. As such, the outcome of our experiments with regards to Cooper’s algorithm is as expected, showing that this algorithm is not usable on medium-sized examples in practice.

## Conclusion and Future Work

We have developed a verified solver for linear mixed integer arithmetic, and have formalized important results on linear integer programming that were needed in order to prove the soundness of the solver. To the extent of our knowledge, the main mathematical theorems of which we formalized proofs had not been previously verified in any formal system, and our solver is the first verified LIA solver that is also usable in practice. The two parts of our formalization amount to 9813 lines of Isabelle code and took roughly 10 person-months to implement.

Currently, our solver is essentially “proof of concept” software, and there are a number of known optimizations that could improve it, e.g., preprocessing of constraints, integration of cutting planes, unit-cube-tests, etc. [6, 7, 14]. We have also used run-time profiling in order to establish which sub-routines our solver spends most time on, and have identified parts of the incremental simplex algorithm that we could further modify in order to improve running times.

## Footnotes

1 The textbook bound is somewhat more precise than ours, as it is phrased in terms of sub-determinants, whereas we use a generic bound on sub-determinants.

2 The Isabelle statement given here matches the presentation of the theorem in [21]; in our formalization, the equivalence is written as an equality of sets.

3 Here, “non-incremental” means that the tests are simply sets of constraints, as opposed to constraints together with an assert/check script that a solver must execute.

4 QF_LIRA (quantifier-free mixed integer real arithmetic) contains only 8 tests.
